# Cancer incidence in urban Shanghai, 1973-2010: an updated trend and age-period-cohort effects

**DOI:** 10.1186/s12885-016-2313-2

**Published:** 2016-04-21

**Authors:** Ping-Ping Bao, Ying Zheng, Chun-Xiao Wu, Zhe-Zhou Huang, Yu-Tang Gao, Fan Jin, Yong-Bing Xiang, Wei-Jian Zhong, Wei Lu, Fan Wu

**Affiliations:** Department of Cancer Control & Prevention, Shanghai Municipal Center for Disease Control and Prevention, Shanghai, 200336 People’s Republic of China; Department of Epidemiology, Shanghai Cancer Institute, Shanghai, 200032 People’s Republic of China

**Keywords:** Cancer incidence, Time trend, Age-period-cohort analysis, Shanghai

## Abstract

**Background:**

To provide a comprehensive overview of temporal trends in cancer incidence during 1973–2010 in urban Shanghai.

**Methods:**

The estimated annual percent changes (EAPCs) for the whole period and for the time segments in age-standardized incidence rates (ASR) were evaluated with Joinpoint analysis. Age-period-cohort (APC) models were modeled to examine the effects of age, period and birth cohort on cancer incidence.

**Results:**

The overall ASR decreased slightly and significantly in males (EAPC of −0.41) but increased significantly in females (EAPC of 0.57) during 1973–2010 in urban Shanghai. The incidence trend was not linear and varied by time segments. During the most recent 10 years (2001–2010), the ASR in males decreased by 1.65 % per year and stabilized in females. Incidence rates continued to decline during 1973–2010 for esophagus, stomach, and liver cancer in both sexes, as well as male lung cancer and cervix cancer. It should be noted that it was the first time to document a significant decline in lung cancer incidence among males during 1973–2010 with EAPC of −0.58 %, and a notable upward for cervix cancer since 1996 with EAPC of 8.94 %. Unfavorable trends in incidence were observed for the most common cancer sites in the 38 years period: colorectum, gallbladder & biliary tract, pancreas, kidney, bladder, brain & central nervous system (CNS), thyroid, non-Hodgkin’s lymphoma (NHL), prostate, female breast, corpus uteri, and ovary. APC analysis showed age, period and birth cohort yielded different effects by cancer sites.

**Conclusions:**

The observed trends primarily reflect dramatic changes in socioeconomic development and lifestyles in urban Shanghai over the past four decades.

## Background

The population-based cancer registry is an indispensable tool for providing data for planning and evaluation of programs for cancer control. There is no national cancer reporting system and a few local cancer registries have collected data on cancer incidence long enough to allow the analysis of sequential trends in China [1]. Sufficiently long period and high-quality population-based data have been collected throughout Shanghai urban areas since 1970s by the Shanghai Cancer Registry (SCR) and published periodically in volumes IV-X of “*Cancer Incidence in Five Continents (CI5)*” by the International Agency for Research on Cancer (IARC) [2, 3]. Cancer incidence trends in urban Shanghai were reported previously [4, 5], however, the data were outdated. Shanghai has experienced rapid changes in aging population, economic development, and social transformation over the past two decades. This will absolutely bring great impact on the cancer patterns and variation trends of cancers.

The current study examined the temporal changes on age-adjusted cancer incidence rates and change points in long-term trends for all cancers combined and for the top 18 site-specific cancers from 1973 through 2010 in urban Shanghai by sex. This report also quantified the effects of age, period, and birth cohort on the observed trends with age-period-cohort (APC) analysis. The comprehensive analysis of cancer variation trends in a long history perspective can not only provide basic information for making the planning and prioritization of prevention activities, but also to spur additional research into the causes of these observed changes.

## Methods

All cancer incidence data were derived from the SCR, an associate member of the International Association of Cancer Registries (IACR). Details of the cancer registry have been previously described [4, 6]. Briefly, the SCR, a population-based cancer registry, systematically collected information on incident cases of cancer in urban Shanghai and the complete incidence data are available from 1973 onward [7]. In 2010, urban Shanghai had a total permanent population of 6.19 million. The SCR has formed standard system to collect, process, and report cancer incidence data. A standardized notification card, which includes information on name, date of birth, sex, address, occupation, primary site of cancer, and date and basis of cancer diagnosis, is used for reporting cancer cases. The completeness of coverage of the Registry is very high with death certification only (DCO) less than 1 %. The data has been published in the last six volumes of *Cancer incidence in Five Continents*. The corresponding population data of Shanghai urban areas were retrieved from the Shanghai Municipal Bureau of Public Security. The study was approved and the need for consent was waived by the institutional review board (IRB) of Shanghai Municipal Center for Disease Control & Prevention. In this study, only data in annual cancer report was used and no information to identify individual subjects was included.

Age standardized rates (ASRs) were calculated by the direct method using the weight of the 1960 world standard population. The estimated annual percent changes (EAPCs), representing the average percent increase or decrease in cancer rates per year over a specified period of time, were obtained using the joinpoint regression. The joinpoint analysis has been widely applied to detect the changes points (joinpoints) and determine the trends between join points, which involves fitting a series of joined straight lines on a logarithmic scale to the trends in annual ASRs [8]. The allowed maximum number of joinpoints was 5 over 38 years as at least 6 years was required for each segment. We used a Joinpoint regression model implemented in the Joinpoint Regression Program (Version 4.0.4) [9].

To further elucidate the trends in incidence rates of all cancer combined and different cancer sites, age-period-cohort (APC) analysis [10, 11] based on Poisson regression for the effect of cohort and period, were undertaken. A Lexis diagram tabulating incidence cases and person-years by age, period and cohort was used for the analysis. The formulation of the multiplicative age-period-cohort model for incidence rates, (*a,p*) at age *a* in period *p* for persons in cohort *c* = *p* - *a*, is: log[*r* (a,p)] = *f* (a) + *g*(*p*) + *h*(c), where the additive effects can be partitioned into linear and nonlinear components [12, 13]. Each of the three functions, f (a), g(p) and h(c), were parameterized using natural splines [12–14]. On adding together the linear and curvature components, the individual categories of each effect were obtained. The relevant sub-models were arranged into a sequence to provide relevant comparisons of linear, non-linear cohort and period effects. Significant curvatures in cohort and period effects was assessed by comparing the differences in the deviances with the degrees of freedom using Chi-square test [11]. The statistical analysis was conducted using the apc.fit implemented on the Epi package [15] from software R version 3.0.1. Statistical significance was attributed to two-sided *P* values <0.05.

For APC analysis of subtypes of cancer, except for esophagus and prostate, an age restriction to ages 20–84 eliminated some of the variation caused by few cases among younger ages and potential biases concerning the accuracy in the oldest age groups. Synthetic twenty 5-year birth cohorts were derived on subtracting the midpoints of the 5-year age groups 20–24, 25–29, 30–34, …, 80–84 from the central year of the 5-year periods of diagnosis 1973–1975, 1976–1980, …, 2006–2010. The mid-point of birth cohort (1937–1941) was set as a reference cohort. For esophagus and prostate, because of the huge variation of scale in APC graphs, we restricted the APC analysis to those aged 30–84 and birth cohorts was obtained by subtracting the midpoints of 5-year age groups from the corresponding calendar year, having the reference cohort of 1932–1936.

### Availability of data

We are unable to share the data owing to the grounds of our ethics approval. Most of data supporting the conclusions of this article is available in the Cancer Incidence in Five Continents (CI5) series: Cancer Incidence in Five Continents Volumes I to X by IARC (http://ci5.iarc.fr/CI5I-X/Default.aspx),

## Results

As shown in Table 1, during the most recent time period, 2006–2010, 122,341 cases of cancer were diagnosed among residents of urban Shanghai. The crude incidence rates for all cancer sites were 414.81 per 100,000 (ASR: 202.13) in males and 378.26 per 100,000 (ASR: 181.76) in females, with a male-to-female sex ratio of 1.10:1. Lung, colorectal, stomach, liver, and prostate cancers were the five top types of cancer among males. Among females, the five most common sites of cancer were breast, colorectal, lung, stomach, and thyroid.

**Table 1:** Temporal trends for cancer incidence rates by sex and selected cancer sites in urban Shanghai, 1973–2010

Sites	ICD-10	Male						Female					
		1973–1977		2006–2010		Percent change^b^	EAPC^c^	1973–1977		2006–2010		Percent change^b^	EAPC^c^
		Count	ASR^a^	Count	ASR			Count	ASR	Count	ASR		
Nasopharynx	C11	662	4.20	811	3.01	−28.38	−0.49	351	2.14	306	1.12	−47.81	−1.71*
Other oral cavity and pharynx	C00-10,C12-14	374	2.63	814	2.71	2.87	0.04	340	2.14	569	1.78	−16.68	−0.42*
Esophagus	C15	3381	25.31	1911	5.62	−77.81	−4.23*	1659	10.48	781	1.60	−84.75	−5.45*
Stomach	C16	8263	59.12	7992	24.20	−59.06	−2.66*	3853	24.05	4681	12.81	−46.74	−1.84*
Colorectum	C18-20	1898	13.58	9252	28.36	108.85	2.37*	1913	11.92	8222	22.33	87.33	2.13*
Liver	C22	5289	34.77	6129	19.94	−42.64	−1.56*	1929	11.91	2459	6.41	−46.17	−1.76*
Gallbladder & biliary tract	C23-24	148	1.12	1020	2.94	162.14	3.15*	280	1.73	1684	3.91	125.73	2.80*
Pancreas	C25	597	4.18	2436	7.33	75.27	1.67*	523	3.31	2174	5.39	62.89	1.61*
Larynx	C32	429	3.12	768	2.39	−23.51	−0.69*	101	0.65	44	0.11	−82.98	−4.88*
Lung	C33-34	6752	48.51	12986	38.13	−21.39	−0.58*	2885	18.02	6667	17.76	−1.47	0.14
Bone	C40-41	304	2.08	237	1.03	−50.38	−2.05*	263	1.64	237	0.95	−41.84	−1.43*
Other skin	C44	237	1.82	651	2.01	10.46	0.50	227	1.45	577	1.59	9.51	0.53
Breast	C50	55	0.36	77	0.25	−29.45	−0.44	2807	17.14	11296	39.51	130.51	2.89*
Cervix	C53	–	–	–	–	–	–	3163	19.02	1271	5.30	−72.16	−3.44*
Corpus uteri	C54	–	–	–	–	–	–	416	2.43	1746	5.86	141.28	3.06*
Ovary	C56	–	–	–	–	–	–	665	4.09	1938	7.10	73.57	2.07*
Prostate	C61	187	1.62	4453	12.17	651.27	6.89*	–	–	–	–	–	–
Kidney & renal pelvis	C64-66	172	1.27	2466	8.41	562.05	6.41*	135	0.93	1305	4.06	336.50	5.42*
Bladder	C67	828	6.49	2609	7.72	18.93	0.82*	318	2.01	889	2.18	8.64	0.59*
CNS	C70-72	494	3.50	1496	6.01	71.77	1.85*	436	3.03	1878	6.49	114.05	2.99*
Thyroid	C73	285	1.80	1045	4.58	154.41	2.77*	850	5.26	3439	14.78	180.96	3.47*
Hodgkin lymphoma	C81	127	0.86	66	0.36	−57.70	−1.71*	82	0.51	32	0.14	−72.33	−2.82*
NHL	C82-85,C96	491	3.43	1409	5.05	47.13	1.64*	284	1.77	1139	3.65	106.48	2.50*
Multiple myeloma	C88 + C90	81	0.52	471	1.47	183.41	3.01*	65	0.38	314	0.83	119.15	2.83*
Leukemia	C91-95	681	4.99	1269	5.47	9.68	0.13	587	4.17	1003	4.23	1.43	−0.17
All sites	C00-95	33629	239.03	64015	202.13	−15.44	−0.41*	26287	163.81	58326	181.76	10.96	0.57*

^a^ASR: age-standardized rates, per 100,000 person-years, directly age-adjusted with the world standard. ^b^Based on unrounded rates. ^c^EAPC: estimated average annual percent change, from Joinpoint analysis. *p ≤0.05

### Trends in ASRs

Table 2 showed the long-term trend and trend patterns for all cancer combined and for the eighteen common cancer sites based on the joinpoint analysis by sex from 1973 to 2010. Among males, incidence rates for all cancers combined slightly but significantly decreased by an estimated annual percent change (EAPC) of −0.41 %. In contrast to males, a statistically significant increase of 0.57 % per year was noted among females, despite the lower average incidence compared with males. For male incidence, there were two joinpoints at 1996 and 2001: the rate decreased by −0.79 % per year from 1973 to 1996, stabilized from 1996 to 2001, and decreased by −1.65 % per year from 2001 to 2010. For females, there were three joinpoints at 1980, 1996 and 2001: the rate decreased by −3.01 % annually from 1973 to 1980, stabilized from 1980 to 1996, increased by 4.38 % annually from 1996 to 2001, then remained unchanged from 2001 to 2010.

**Table 2:** Time period for temporal trends in age-adjusted cancer incidence for selected cancers by sex in Shanghai, 1973–2010

		Trend 1		Trend 2		Trend 3		Trend 4		Trend 5	
	EAPC\(%)^a^	Years	EAPC(%)	Years	EAPC(%)	Years	EAPC(%)	Years	EAPC(%)	Years	EAPC(%)
All sites											
Male	−0.41*	1973–1996	−0.79*	1996–2001	2.29	2001–2010	−1.65*				
Female	0.57*	1973–1980	−3.01*	1980–1996	0.30	1996–2001	4.38*	2001–2010	−0.14		
Esophagus											
Male	−4.23*	1973–1994	−5.14*	1994–2001	−0.39	2001–2010	−6.58*				
Female	−5.45*	1973–2010	−5.45*								
Stomach											
Male	−2.66*	1973–1989	−1.67*	1989–1995	−5.36*	1995–2000	0.44	2000–2010	−4.23*		
Female	−1.84*	1973–1989	−1.00*	1989–1995	−3.84*	1995–2000	1.16	2000–2010	−3.88*		
Colorectum											
Male	2.37*	1973–2010	2.37*								
Female	2.13*	1973–1995	2.15*	1995–2002	4.18*	2002–2010	−1.65*				
Liver											
Male	−1.56*	1973–1996	−1.77*	1996–2002	0.79	2002–2010	−3.97*				
Female	−1.76*	1973–2010	−1.76*								
Gallbladder & biliary tract											
Male	3.15*	1973–2000	4.44*	2000–2010	−2.31						
Female	2.80*	1973–1987	5.53*	1987–1995	1.87	1995–2000	6.68	2000–2010	−3.72*		
Pancreas											
Male	1.67*	1973–1982	4.00*	1982–2010	1.25*						
Female	1.61*	1973–2010	1.61*								
Lung											
Male	−0.58*	1973–1989	0.79*	1989–1994	−2.93	1994–1999	2.52	1999–2010	−3.45*		
Female	0.14	1973–1995	−0.29	1995–2001	3.07	2001–2010	−1.89*				
Kidney & renal pelvis											
Male	6.41*	1973–1981	−1.03	1981–2010	7.54*						
Female	5.42*	1973–1980	−4.48	1980–2010	6.60*						
Bladder											
Male	0.82*	1973–2010	0.82*								
Female	0.59*	1973–1991	−0.89	1991–2010	1.97*						
CNS											
Male	1.85*	1973–1980	−1.09	1980–1989	5.57*	1989–2010	0.52				
Female	2.99*	1973–1980	−2.28	1980–1985	8.54	1985–2003	3.31*	2003–2010	−1.67		
Thyroid											
Male	2.77*	1973–1978	0.85	1978–1983	−15.81*	1983–2001	3.60*	2001–2010	18.10*		
Female	3.47*	1973–1984	−9.58*	1984–2002	5.57*	2002–2010	17.72*				
NHL											
Male	1.64*	1973–2010	1.64*								
Female	2.50*	1973–2010	2.50*								
Leukemia											
Male	0.13	1973–2010	0.13								
Female	−0.17	1973–1997	−1.09*	1997–2010	2.13*						
Prostate	6.89*	1973–1989	0.51	1989–2004	12.62*	2004–2010	3.59				
Female breast	2.89*	1973–1981	−0.17	1981–1990	4.45*	1990–1996	0.77	1996–2001	7.91*	2001–2010	0.23
Cervix	−3.44*	1973–1980	−19.07*	1980–1996	−6.99*	1996–2010	8.94*				
Corpus uteri	3.06*	1973–1996	2.52*	1996–2001	10.24*	2001–2010	−2.00				
Ovary	2.07*	1973–1984	−0.55	1984–2005	3.50*	2005–2010	−5.66				

^a^EAPC: estimated annual percent change in age-adjusted incidence rates, from join point analysis using Joinpoint software. *Estimated annual percentage change (EAPC) significantly different from zero (*P* < 0.05)

Throughout the whole 38-year time period, the incidence rates had increased steadily since 1973 for 5 common cancer sites: pancreas (EAPC: 1.67 % for males, 1.61 % for females), kidney & renal pelvis (EAPC: 6.41 % for males, 5.42 % for females), bladder (EAPC: 0.82 % for males, 0.59 % for females), non-Hodgkin lymphoma (NHL) (EAPC: 1.64 % for males, 2.50 % for females), and colorectal cancer in males (EAPC, 2.37 %). Unfavorable trends in incidence were also observed for gallbladder & biliary tract (EAPC: 3.15 % for males, 2.80 % for females), brain & central nervous system (CNS) (EAPC: 1.85 % for males, 2.99 % for females), thyroid (EAPC: 2.77 for males, 3.47 % for females), colorectal in females (EAPC: 2.13 %), corpus uteri (EAPC: 3.06 %), and ovary (EAPC: 2.07 %) cancers during the observed period. The rates for these cancers, except for thyroid cancer, reached a peak and had declined or stabilized in the most recent 10 years. Trends pattern in thyroid cancer was more complex- decreasing before early 1980s at an annual rate of −15.81 % in males and −9.58 % in females, then increasing afterwards with a larger rate in the most recent 10 years accelerating to 18.10 % in males and 17.72 % in females, respectively.

In contrast, continuous decreases in incidence rates of esophagus (EAPC: −4.23 % for males, −5.45 % for females), stomach (EAPC: −2.66 % for males, −1.84 % for females), and liver (EAPC: −1.56 % for males, −1.76 % for females), cancers were observed, with almost no difference between males and females.

Lung cancer remained the most common cancer in males, while a slight but significant decrease at an annual rate of −0.58 % during 1973–2010 was seen. Male lung cancer tended to decline substantially in the most recent 10 years at an annual rate of −3.45 %. Among females, although the incidence was relatively stable over the whole period, the rates in females showed signs of decreasing since 2001 with an EAPC of −1.89 %. Regarding the trends for leukemia, the incidence appeared to be steady over the years, which seemed rather stable in male. For females, the rates had increased steadily since 1997 at an annual rate change of 2.13 %.

There had been a marked increase in incidence for prostate cancer during the four decades with EAPC approaching 6.89 %. The very sharp increase (EAPC 12.62 %) detected between 1989 and 2004 seemed to have slowed down in recent years. Trends in incidence from female breast cancer and cervical cancer were different with an increase of 2.59 % per year and a decline of −3.44 % per year from 1973 through 2010. Female breast cancer was upward before 2001 with a sharp increase by 7.91 % per year in 1996–2001 and then reached a plateau during the recent 10 years. The decline in incidence for cervix cancer was steep until 1996, when trends reversed and significantly increased by 8.94 % per year.

### Age-period-cohort analysis for selected cancers

**Fig. 1:**
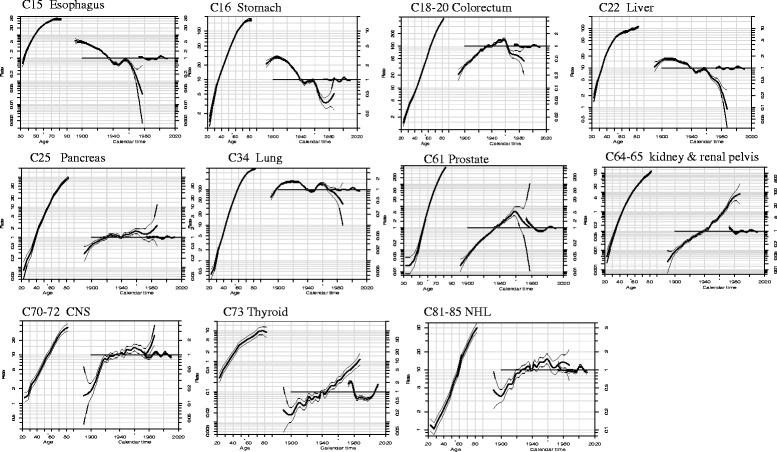
Estimated Age-Period-Cohort effects for incidence of selected cancers in urban Shanghai (1973–2010) among males Note: Age-period-cohort effects, with confidence limits, on incidence from common cancer sites in urban Shanghai. Each graph has three curves depicting, from left to right, trends in incidence rate by age for the reference cohort (age effect), incidence risk by birth cohort (cohort effect,taking 1937–1941 or 1932–1936 as the reference) and incidence risk by calendar year (period effect, taking the incidence average of the period as the reference). The graph has the horizontal axis divided into two parts: one for age (years old) and one for cohort-period (calendar years). The left vertical axis represents incidence rates for the age effect and the right vertical axis represents the relative risk for the cohort and period effect. The drift is added to the non-linear birth cohort effects and the right plot presents the period effect as residual ratio rates

**Fig. 2:**
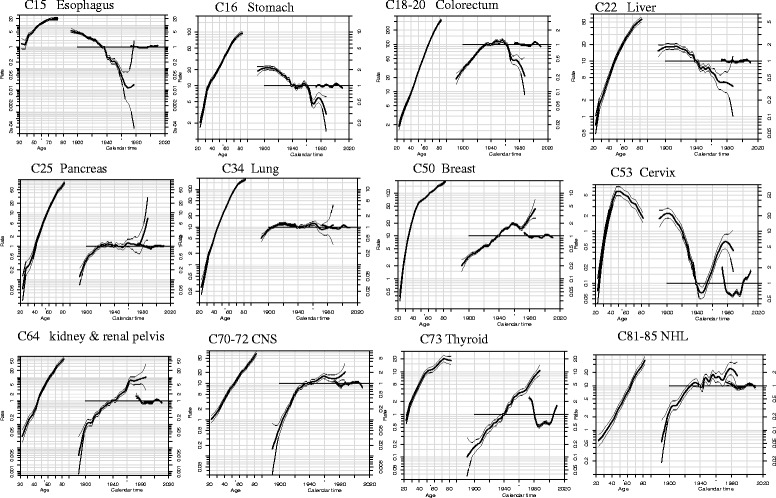
Estimated Age-Period-Cohort effects for incidence of selected cancers in urban Shanghai (1973–2010) among females. Note: Age-period-cohort effects, with confidence limits, on incidence from common cancer sites in urban Shanghai. Each graph has three curves depicting, from left to right, trends in incidence rate by age for the reference cohort (age effect), incidence risk by birth cohort (cohort effect, taking 1937–1941 or 1932–1936 as the reference) and incidence risk by calendar year (period effect, taking the incidence average of the period as the reference). The graph has the horizontal axis divided into two parts: one for age (years old) and one for cohort-period (calendar years). The left vertical axis represents incidence rates for the age effect and the right vertical axis represents the relative risk for the cohort and period effect. The drift is added to the non-linear birth cohort effects and the right plot presents the period effect as residual ratio rates

The effects of age, period and cohort on incidence rates by cancer sites according to sex were displayed in Figs. 1 and 2.

Three main patterns of evolution were observed for cohort effects: the first group with four cancer sites (esophagus, stomach, and liver cancers) in both sexes, in which incidence trends were consistent downward throughout all birth cohorts; the second group comprised of colorectum in male, prostate, kidney & renal pelvis, NHL, thyroid, female breast, ovary, and corpus uteri cancers, in which all successive birth cohorts experienced a steady rise in the risk of developing cancer, with no clear evidence of downward trend for the younger generations; the third group with colorectum in female, gallbladder & biliary tract, pancreas, bladder, and CNS cancers, in which incidence fluctuated in all birth cohorts and showed a upward trend generally.

The lung cancer incidence rates in both sexes were accelerated in birth cohorts born in the early 1900s, and then declined in 1930, followed with further accelerations in about 1945 and deceleration rates in the 1960s. A different pattern of birth cohort effect of cervix cancer was observed: a declining risk till later 1940s, then a steady rising risk in every later consecutive birth cohorts.

During the period 1973–2010, the trend of period effect was oscillating in each of cancer sties. The period rate ratios remained stable for most of common cancer sites (esophagus, stomach, colorectum, liver, pancreas, lung, kidney & renal pelvis, bladder, CNS, NHL, leukemia, prostate, female breast, corpus uteri, and ovary). APC models showed a strong period effect in both sexes for thyroid cancer, which decreased through the early 1980s, then increased thereafter. The period effect in cervix cancer also yielded significant transitions in incidence trends: a falling trend up to the mid-1990s and a rising trend after the early 2000s.

## Discussion

This study provided a comprehensive and up-to-date overview of temporal trends and the age-period-cohort effects on cancer incidence in urban Shanghai over the past four decades. Regarding trends for all cancers combined, the incidence declined slightly and significantly in males during the whole period of 1973–2010, but a statistically significantly increased trend was noted in females. During the most recent 10 years (2001–2010), the overall cancer incidence rates in males decreased by 1.65 % per year, but stabilized in females. The results showed that the trends and change points for incidence trends varied by cancer sites, and the age, period and birth cohort also yielded different effects by cancer types. The trend patterns could be predicted by both time periods of diagnosis and birth cohort components. The cancer types which were common among males and females had similar trends and the trends had no significant difference according to EAPC values, except for lung cancer.

Scrutiny of trends in cancer incidence can help to recognize trends related to changes in cancer risk, including risks mediated by exposures to environmental and occupational carcinogens. With its diversity as an asset, Shanghai has enjoyed remarkable economic growth in the last four decades, experiencing rapid changes in population aging, urbanization, and social transformation, which could bring great impact on cancer patterns and variation of temporal trends. The particular tumors selected that may be influenced by these changes were discussed below.

Esophagus and stomach cancers showed a remarkable and steady decrease in incidence over the study period, and the trends were also reflected in the gradual decline in risk in successive generation among both sexes born approximately after 1900. The decrease in esophageal and stomach cancer rates in both males and females can be expected to continue in consideration of the sharp decline in younger birth cohorts. The patterns were likely to be attributable to the general improvements in living conditions in Shanghai, such as increasing consumption of fresh fruit and vegetables, the widespread use of refrigeration, and decreasing intake of salt-preserved food and beverage at high temperatures [5, 16]. Other possible determinants for the favorable trends of stomach cancer were reductions in infection of *Helicobacter pylori* because of improvements in socioeconomic status and sanitary conditions of housing [5].

Colorectal cancer was one of the most rapidly increasing malignancies and it was the second most frequent cancer in urban Shanghai. Previous studies have suggested that maintenance of a healthy diet and avoidance of a sedentary lifestyle and obesity may reduce the risk of colorectal cancer [17]. It thus appeared that the changes in lifestyles, such as less physical activity, and excess weight, may contribute to the persistently rising rates in urban Shanghai [18, 19]. In the US, continuous decline in colorectal cancer incidence in recent years was most likely attributable to significant improvements in the use of colorectal cancer screening [20]. A local colorectal cancer screening program using biennial fecal occult blood testing (FOBT), followed by total colonoscopy in positive patients, has been implemented in Shanghai since 2012, and this program will influence its incidence trend in future.

Incidence from liver cancer was favorable for both sexes over the whole period and the declines in risk have been observed among successive birth cohorts born since 1915s in urban Shanghai. Etiological determinants of liver cancer include chronic infection with hepatitis B virus (HBV), and exposure to dietary aflatoxin, alcohol consumption and smoking [21, 22], all of which may partially contribute to the decreasing trend. Measures to prevent HBV infection by managing blood and its products, and measures to ensure an improved water quality by purifying polluted water source, have largely decreased the risk of HBV infection and aflatoxin B1, respectively [23]. Other measures such as attempts to reduce the consumption of smoking and heavy drinking, and a focus on a balanced diet [24, 25] may also be attributed to the risk downward of liver cancer. The newborns in urban Shanghai have been inoculated with the HBV vaccine under the aggressive vaccination program since 1992 [25], so further decreased incidence will be seen in these children as they become adults about 10–30 decades later [26]. It should be noted that liver cancer incidence rates were increasing in many parts of the world including the US and Central Europe, possibly due to the obesity epidemic and the rise in hepatitis C virus (HCV) infection [27].

The incidence of pancreas cancer in urban Shanghai has increased constantly since the 1970s, which is consistent with the increasing rates among successive birth cohorts. Factors that contributed to the increasing rates in males and females were not fully elucidated. Limited studies showed that improvement in the diagnosis of pancreas cancer and changes in lifestyles (such as changing of dietary patterns and increasing prevalence of obesity) may partly explain the rising trend [28–31].

This study was the first to document a significant slight decrease in lung cancer incidence among males during the observed period in urban Shanghai, with accompanying more substantial decline in the most recent 10 years, a changing from a plateau to a gradual reduction. Similarly, although the incidence was relatively stable in females over the whole period, an encouraging trend after 2001 was observed. APC models also suggested that incidence rates began to decline for younger generations born around 1960. Therefore, the decreases in lung cancer rates in both males and females can be expected to continue with lower risk of younger birth cohorts. Overwhelming majority of lung cancer was caused by tobacco smoking, accounting for 80 % of the worldwide lung cancer burden in males and at least 50 % of the burden in females [27]. The prevalence of smoking among males tripled in China from the early 1950s to the late 1980s and reached 61 % in Shanghai during 1970s [5]. The smoking prevalence in Shanghai showed signs of stability or slight decreasing since 1970s and estimated as 55.37 % among males and 1.47 % among females aged 15 and above in 2010 [32]. Lung cancer risk among women in urban Shanghai was linked to smoking, as well as to exposure to rapeseed oil vapors during high-temperature cooking [33]. The downward trend observed in female lung cancer incidence in recent 10 years reflected the improvements in kitchen ventilation and in living conditions. The risk of lung cancer from cigarette consumption among males in Shanghai may have been attenuated by some protective factors such as intake of certain fruits and vegetables and by improvements in the work environment [5, 34].

Female breast cancer has increased substantially in urban Shanghai, becoming the leading cancer among females in the late 1980s. Population-based breast cancer screening programs with effective tools such as mammography and ultrasonography have yet to be implemented in Shanghai. This substantial increase trend in urban Shanghai was driven by cohort effects. Menstrual and reproductive factors, such as early age at menarche, late age at first pregnancy, lack of lactation, and decreasing number of children because of one-child-per-family policy, may partially explain the increasing trend of breast cancer [4, 35]. Other factors including changes in lifestyles, such as high consumption of animal foods and fat, less physical activity, and obesity, may be related to the rising incidence [16, 36]. In contrast, the burden of cervical cancer substantially decreased over time. Strong period and cohort effects were observed in its trend. The dramatic decline in cervix cancer before 1996 could be largely attributed to the widespread Pap smear screening and treatment programs first implemented in Shanghai during the late 1950s [5, 37]. Notably, there appeared a rising trend since 1996. Higher prevalence of Human Papilloma virus (HPV), and changes in lifestyles and sexual behaviors can be partially attributed to the rising risk among younger generations [38].

During the past four decades in urban Shanghai, prostate cancer incidence rates have increased substantially. The incidence rate was among the lowest in the world at 1970s and it became the fifth most common cancer in males after 2002. APC analysis showed that cohort effect increased steadily after the year corresponding to the minimum risk value, whereas period effect seemed to remain unchanged throughout the years. There has been no national or local prostate cancer screening in Shanghai and the reasons for the upward trend may suggest a role of environmental factors and/or increased detection of the tumors based on improved technology and surveillance [4].

Thyroid cancer incidence decreased through the early 1980s, then increased thereafter. The results from APC modeling showed that both period and birth cohort effects appeared to have an impact on the observed incidence trends. The downward during the beginning 10 years may be explained in part by the mass screening conducted in several factories in the mid-1970s, which detected large numbers of occult cancer cases in that period [4]. The improvement in diagnostic modalities and increased medical detection of small thyroid nodules may contribute to the unusual increase in the incidence during the last two decades in both sexes. However, the effects of iodine supplementation and some birth cohort-related changes in environmental exposures (such as increased exposure to diagnostic X-rays) cannot be ruled out for the observed increased risk of thyroid cancer among young birth cohorts. Same patterns in incidence of thyroid cancer were found in the US and Denmark [39, 40]. Further detailed analysis based on histopathology is needed to clarify the potential contributors for the sharp upward of incidence.

Over the study period, the incidence of kidney & renal pelvis cancer had risen rapidly since the 1970s. Accumulating evidences have suggested an etiologic role in renal cell carcinoma for physical activity, alcohol consumption, and occupational exposure to trichloroethylene [41]. Among malignancies of the hematopoietic system, a steady increase in incidence was observed for NHL. No clear understanding of the risk factors can explain the long-term trends. Increasing exposure to one or more ubiquitous lymphomagenic agents [42] such as pesticides may partly contribute to the upward in incidence. Further researches are warranted to clarify the potential risk factors for these cancers with increasing incidence in urban Shanghai.

There were no substantial changes in the registry operation occurred over the study period. It was plausible that at least part of the observed trends on cancer incidence may reflect changes in the prevalence of risk factors and the measures taken for cancer prevention and control in the population. The incidence trends for all cancers combined for males and females varied. The significant download trend in males may be partly explained by the declines in several cancer rates, including esophagus, stomach, liver, and lung cancer, while the increased incidence of colorectum, breast, and thyroid cancers could be partly attributed to the upward trend in females.

## Conclusions

In summary, the overall ASR decreased slightly and significantly in males but increased significantly in females from 1973 to 2010 in urban Shanghai. The incidence trends were not linear for all cancers combined and for common cancer sites and varied by time segments. The observed trends could reflect dramatic changes in socioeconomic development and lifestyle in urban Shanghai over the past four decades and underscore the need for additional prevention efforts for the cancers with constant increasing incidence or rising again.

## Abbreviations

APC: age-period-cohort
ASR: age-standardized incidence rates
CNS: brain & central nervous system
EAPC: estimated annual percent changes
HBV: hepatitis B virus
NHL: non-Hodgkin’s lymphoma
SCR: Shanghai Cancer Registry
